# Creep Behavior of Poly(lactic acid) Based Biocomposites

**DOI:** 10.3390/ma10040395

**Published:** 2017-04-08

**Authors:** Marco Morreale, Maria Chiara Mistretta, Vincenzo Fiore

**Affiliations:** 1 Faculty of Engineering and Architecture, Kore University of Enna, Enna 94100, Italy; 2 Department of Civil, Environmental, Aerospace, Materials Engineering, University of Palermo, Palermo 90128, Italy; vincenzo.fiore@unipa.it (V.F.); mariachiara.mistretta@unipa.it (M.C.M.)

**Keywords:** biocomposites, PLA, flax, jute, creep

## Abstract

Polymer composites containing natural fibers are receiving growing attention as possible alternatives for composites containing synthetic fibers. The use of biodegradable matrices obtained from renewable sources in replacement for synthetic ones is also increasing. However, only limited information is available about the creep behavior of the obtained composites. In this work, the tensile creep behavior of PLA based composites, containing flax and jute twill weave woven fabrics, produced through compression molding, was investigated. Tensile creep tests were performed at different temperatures (i.e., 40 and 60 °C). The results showed that the creep behavior of the composites is strongly influenced by the temperature and the woven fabrics used. As preliminary characterization, quasi-static tensile tests and dynamic mechanical tests were carried out on the composites. Furthermore, fabrics (both flax and jute) were tested as received by means of quasi-static tests and creep tests to evaluate the influence of fabrics mechanical behavior on the mechanical response of the resulting composites. The morphological analysis of the fracture surface of the tensile samples showed the better fiber-matrix adhesion between PLA and jute fabric.

## 1. Introduction

In the last decades, lignocellulosic fibers have been receiving growing attention both from the academia and the industry, as possible substitutes of synthetic fibers as reinforcement for polymer-based composite materials. This is mainly due to their adequate specific mechanical properties, low cost, good insulation properties, lower health-related issues and recyclability [1]. There is a wide range of different natural fibers that are used as reinforcement of polymer based composites such as wood, flax, jute, hemp, kenaf, ramie and sisal [2,3,4,5]. In particular, flax (*Linum usitatissimum*) is nowadays one of the most widely utilized natural fibers [6,7,8,9,10] and was one of the first to be extracted, spun and woven into textiles [11]. Several industrial fields focused in the last years their attention towards flax fiber thanks to its specific mechanical properties (i.e., comparable with those of E-glass fiber [12]) and sound absorbing efficiency. On the other hand, jute is the second most important fiber in terms of world production levels of cellulosic fibers, next to cotton [13]. The tensile strength, Young’s modulus and elongation at break of jute fiber have been reported to be in the ranges 393–773 MPa, 13–26.5 GPa and 7%–8%, respectively, whereas its density is between 1.3 and 1.45 g/cm^3^ [14]. Nevertheless, the use of conventional oil-based polymers as matrices leads to high environmental impact during the production process, service life, life-end and disposal phases of these composites. For this reason, biodegradable matrices obtained from renewable sources such as poly(lactic acid) (PLA) were considered [15,16,17,18,19,20,21,22,23]. The addition of natural fibers into PLA matrix leads to composites having relatively low price and high stiffness, maintaining an environmental advantage [24,25,26,27]. The mechanical behavior of PLA composites reinforced with vegetal fibers has been widely investigated by several researchers [28,29,30,31,32,33] even if, at the best of our knowledge, few papers deal with the creep behavior of these kinds of biocomposites. 

Yang et al. [34] studied the short-time creep resistance of short bamboo fiber reinforced recycled PLA composites, upon varying the fiber weight content from 5% to 80%. In detail, isotherm creep and creep recovery cycles were conducted in flexural mode by varying the temperature in the range from 20 to 50 °C. For each test, the stress level was set equal to 30% of the average flexural strength for 1 h, followed by a 1 h recovery period. The results revealed that the composites with 60 wt % fiber exhibited the best creep resistance, and then decreased when the fiber loading was higher than 70 wt %, thus evidencing that the addition of bamboo fiber to neat PLA significantly improved creep resistance, due to the better stiffness and heat conduction of bamboo fiber. 

The short-time creep behavior of composites based on a biodegradable polymer matrix (i.e., a blend of poly(butylene adipate-terephthalate) (PBAT) copolyester and PLA) reinforced with three different wood fiber types (at 20 and 30 wt %) were studied in tensile and single cantilever mode [35]. Creep tests were performed for 1 h, at three different temperatures (i.e., 25, 35 and 45 °C), at three stress levels (1, 2.5 and 4.5 MPa for the tensile mode, 4.5, 7 and 10 MPa for the flexural one). In all cases, it was found that wood fibers improved the creep resistance of the composites. 

Some research activities were addressed to the modification of the PLA matrix to enhance the fiber-matrix compatibility, thus leading to an increase in the creep resistance of the resulting composites. In this context, a positive effect of fiber surface treatments (i.e., by means of silane coupling agent and polyvinyl alcohol) on the flexural creep properties of jute reinforced PLA composites was found by Takemura et al. [36]. 

Rozine et al. [29] compared the tensile creep behavior of PLA based composites reinforced with short flax and viscose (i.e., made of regenerated cellulose extruded from a solution of viscose) fibres. In particular, creep tests was performed by varying the stress level (i.e., 12, 15 and 18 MPa) and relative humidity (i.e., 34 and 66%) at room temperature in alternative mode (i.e., alternate steps of creep load and strain recovery). Similarly, Varna et al. [37] analyzed the viscoplastic and viscoelastic strain development of PLA reinforced with short flax fibers performing creep and strain recovery tests at several high stress levels. 

Aim of the present paper is to evaluate the viscoelastic response of PLA reinforced with natural woven fabrics, focusing on the effect of temperature and fabric used as reinforcement (i.e., flax and jute). The effect of fabric on the behavior of PLA biocomposites was also evaluated by performing quasi-static and dynamic mechanical tests, both carried out in tensile mode. Furthermore, scanning electron microscopy (SEM) was used to investigate the fracture surface morphologies of the tensile samples in order to deepen the investigation on fiber-matrix adhesion.

## 2. Results

### 2.1. Tensile Tests

**Figure 1:**
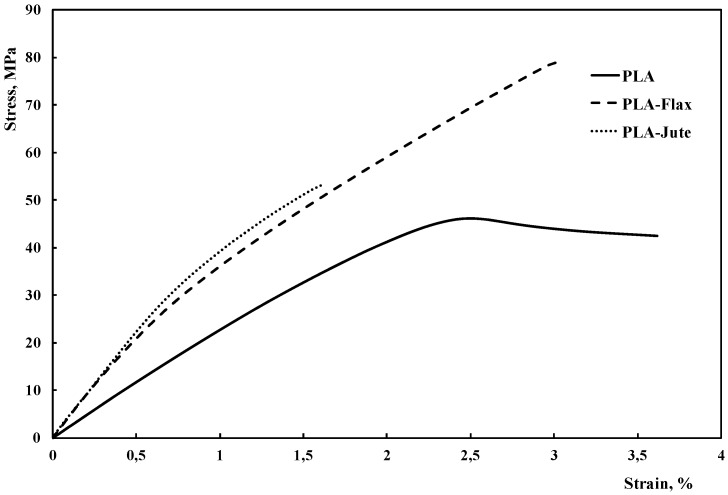
Stress-strain curves of PLA and related composites.

The results from the tensile tests are reported in Table 1, while some representative stress-strain curves are shown in Figure 1.

As concerns composite structures, the results clearly show that the presence of the fabrics leads to a significant increase of the modulus (almost twofold) in comparison to the neat PLA. This can be easily explained considering the stiffening effect due to the fabrics. However, only a marginal variation of the ductility occurs (except, although only partially, for the jute-containing composite). This suggests a good fiber-matrix adhesion. Furthermore, the presence of the flax fabric also leads to a significant increase of the tensile strength (up to 53%), while the jute fabric fails in imparting noteworthy improvements. This result is rather unexpected, and may be explained considering the reduction of the elongation at break [38], as further observable on the basis of the representative stress-strain curves shown in Figure 1. It can be also noticed that the presence of the fabrics modifies the fracture behavior, from a moderately ductile to a completely fragile one [15]. 

The tensile characterization performed on the fabrics as received clearly points out that the flax fabric presents a remarkably higher tensile resistance in comparison to jute fabric (tensile strength approx. 140% higher). However, this dramatic difference, although influencing the composites tensile strength, does not lead to a likewise increase of the PLA-Flax tensile strength in comparison to PLA-Jute. This might be due to a different degree of interfacial adhesion between the matrix and the fabric, and needs further investigation as follows. 

### 2.2. Dynamic Mechanical Thermal Analysis (DMTA)

**Figure 2:**
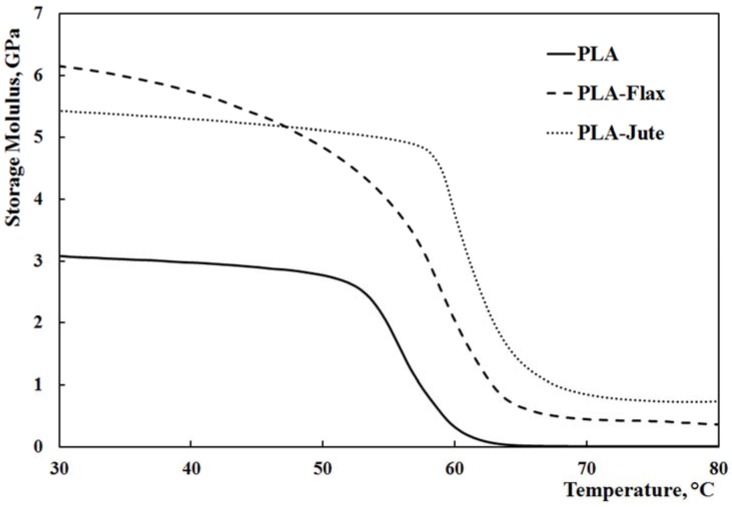
Storage modulus *E’* versus temperature trends.

**Figure 3:**
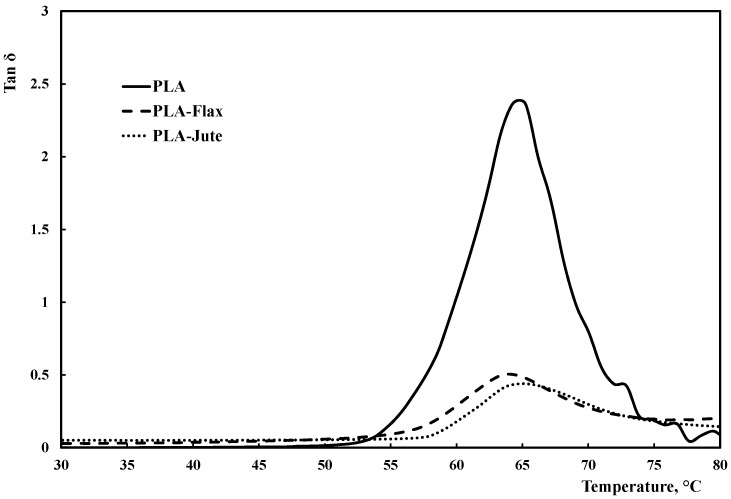
Tanδ versus temperature trends.

Figure 2 reports the storage modulus, *E*’, as a function of the temperature, while Figure 3 shows the corresponding trend of the damping factor, Tanδ. The modulus of the composites is significantly higher than the PLA one, throughout the entire temperature range; furthermore, PLA-Jute composites show an enhanced thermomechanical stability, as clearly observable by considering the lower slope of the curve upon increasing the temperature. This can be further observed by taking into account the modulus at 30 °C (which is, basically, the room temperature) in relationship to the temperature at which the modulus is half of the initial value (*T*_1/2_, halving temperature), see Table 2. In addition to the composites showing higher halving temperatures than the neat PLA, the presence of jute fabrics as reinforcement leads to a slight increase of the halving temperature (approx. +6%), i.e., to an increase of the thermomechanical resistance of this composite. More considerations can also be drawn by considering the glass transition temperature, *T*_g_, and the height of the damping (Tanδ) peak (Table 2). While the *T*_g_ values, calculated as the temperature at which the damping attains its maximum value [39,40], do not change much with the presence of the reinforcing fabrics, significant variations of the damping can be observed. Incorporation of fillers in a composite system typically affects the damping behavior, due to shear stress concentrations in the fibers along with viscoelastic energy dissipation in the matrix [39,41]. The filler–matrix adhesion directly affects Tanδ, i.e., a weak filler–matrix adhesion leads to higher Tanδ values, while a good filler–matrix adhesion hinders the mobility of the polymer chains, thus reducing the damping [41,42,43]. Furthermore, a low damping typically means that the composite has a good load bearing capability. The experimental results shown in Table 2 and Figure 3 clearly point out that the damping undergoes a dramatic decrease upon combining PLA with the fabrics, in comparison to the neat PLA. In addition, the jute-containing composite has an approx. 20% lower damping, in comparison to the flax-containing one. This suggests that the jute fabric leads to a composite with a higher load-bearing capability and a likewise stronger adhesion with the PLA matrix. This result is in agreement with the results from tensile tests reported above: In fact, the better adhesion between jute and PLA leads to a lower difference (i.e., about 50%) in terms of tensile strengths of the two composites, in comparison with the one shown by the two fabrics (about 140%).

### 2.3. Creep Tests

**Figure 4:**
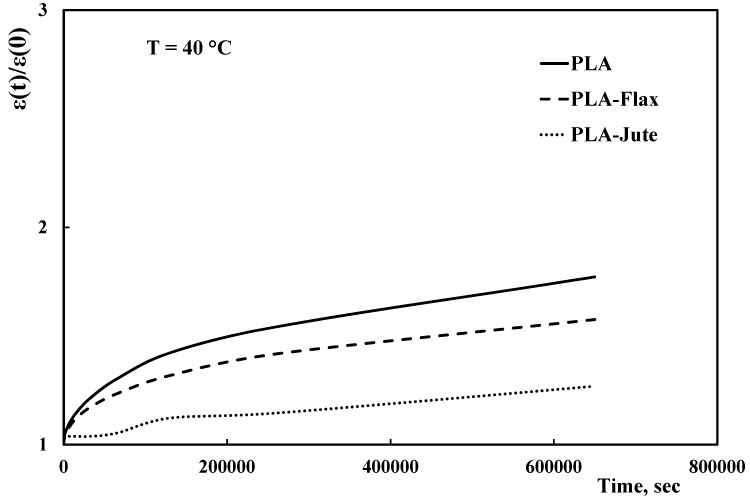
Normalized deformation versus time trends of PLA and related composites at 40 °C.

Figure 4 shows the normalized creep curves for the neat PLA and the related composites, obtained at 40 °C. Normalization was carried out by dividing the actual deformation value at time t, ε(*t*), against the deformation at test start (*t* = 0), ε(0); this allows to better assess the actual differences in creep behavior between the investigated materials. 

With regard to the neat PLA, the creep curve shows a typical trend with primary (decreasing slope) and secondary (approx. constant slope) creep behavior. The presence of the fabrics evidently changes the behavior, leading to significant decreases of the deformation values. In particular, the jute fabric allows to better reduce the creep behavior of the neat PLA in comparison to flax one. Furthermore, the jute fabric leads to only marginal creep deformations. 

Table 3 reports the ratios between the normalized deformation of each composite and the normalized deformation of the neat PLA at specific times (i.e., 8,000, 65,000, 250,000 and 650,000 s). These values allow evaluating the actual reduction of the creep deformation due to the fabrics. 

The best overall creep behavior of the composite containing jute fibers is clearly evidenced taking into account that, after 65,000 s at 40 °C, the creep deformations of the flax and jute composites are respectively 3% and 19% lower than that of the neat PLA. This difference increases upon increasing the test time so that, at the end of the creep tests (i.e., after 650,000 s), the creep deformation reductions of the flax and jute composites are 6% and 28% lower than that of the neat PLA, respectively.

**Figure 5:**
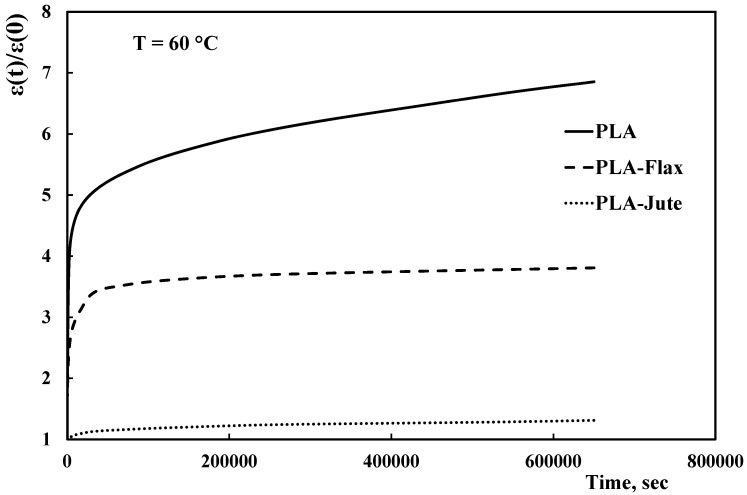
Normalized deformation versus time trends of PLA and related composites at 60 °C.

Figure 5 reports the normalized creep curves at 60 °C. The temperature increment of 20 °C leads to a dramatic increase of the creep deformations. More in details, the PLA normalized deformation increases from values below 2, to values near to 7, and this can be explained by considering that the test temperature is very close to the glass transition temperature (i.e., about 64 °C, see Table 2). The presence of the flax fabric leads to a drastic reduction of the creep deformation (see Table 3). After 65,000 s, the creep deformation of the flax composites is the 66% of that of the neat PLA (56% at the end of the creep test). However, the most relevant result is obtained by the jute-containing composites. As a matter of fact, the presence of jute fabric leads to a creep curve with a nearly-constant trend, very close to 1: This indicates that creep (viscous) deformations are practically neutralized by the jute fabric, leading to an almost completely elastic behavior. It is worth noting from Table 3 that the creep deformation showed by the jute composite remains about 80% lower than that of the neat PLA, during the entire creep test. This means that a remarkable result is obtained: at relatively higher temperatures, the creep deformations of PLA are almost neutralized by the presence of the jute fabrics.

Since the polymer matrix, the manufacturing technique, the fibers nature and the architecture of the fabrics are the same, the striking difference in the behavior between the two composites can be only attributed to a) differences in the creep behavior of the fabrics themselves; b) different fabric-matrix adhesion; c) both of the previous factors. 

**Figure 6:**
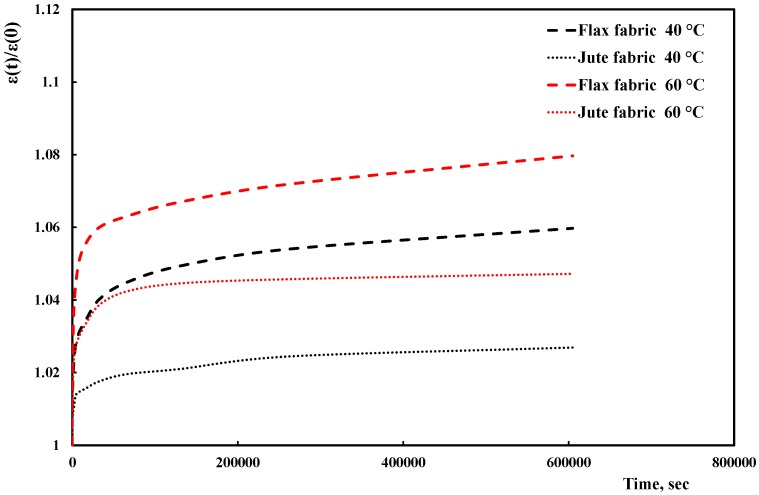
Normalized deformation versus time trends of fabrics at 40 °C and 60 °C.

In order to deeper investigate this issue, creep tests (at the same temperatures as before) were performed on the fabrics as received, and the results are reported in Figure 6. 

The experimental trends clearly point out that, although the jute fabric undergoes smaller creep deformations in comparison to the flax one, the absolute differences between the two fabrics are very limited and obviously lower than those experienced, under the same test conditions, by the respective composites. This suggests that, of the above listed factors, the differences in the creep behavior of the fabrics play a marginal role in determining the overall creep behavior of the composites. Therefore, the major influence on the overall behavior is played by the different fiber-matrix adhesion, which has been already discussed in relationship with the differences in tensile and dynamic-mechanical behavior shown by the two composites. This was further proved by performing a morphological analysis on the fracture surfaces of the composites.

### 2.4. Morphological Analysis

**Figure 7:**
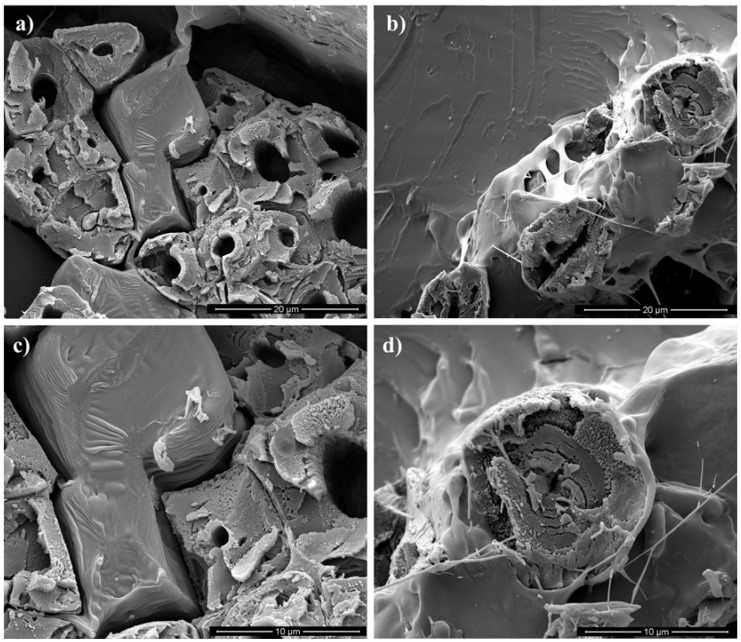
Fracture surfaces of tensile specimens. (**a**) PLA/Flax and (**b**) PLA/Jute at lower magnification; (**c**) PLA/Flax and (**d**) PLA/Jute at higher magnification.

SEM analysis was performed on jute and flax composite samples fractured under tensile quasi-static load in order to provide information on the failure mode and, therefore, on the interface behavior during breaking. Figure 7a–d, show the cross-section fracture surfaces of flax and jute composites, at different magnifications. 

Figure 7a,b allow directly comparing flax and jute composites, respectively. It can be observed that the fiber-matrix adhesion is poor in the flax composite, as shown by the presence of deep gaps and voids at the interface between the polymer matrix and the lignocellulosic fibers. On the other hand, the jute composite has a strongly better adhesion between matrix and fiber, as the wetting of the fibers by the polymer clearly shows. This can be better observed at higher magnifications (Figure 7c,d, respectively). 

**Figure 8:**
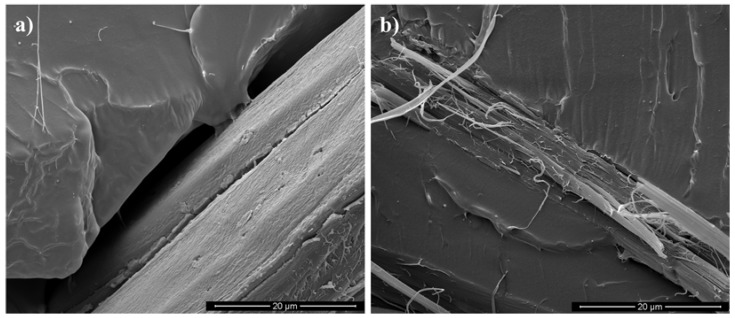
Fracture surfaces showing transverse fibers-polymer matrix interface: (**a**) PLA/Flax and (**b**) PLA/Jute.

However, since the fabric has not only longitudinal fibers (i.e., perpendicular to the fracture surface) but also transverse fibers (parallel to the fracture surface), the comparison between the two composites is also reported in Figure 8a,d, which better show the interface between the polymer matrix and the transverse fibers.

Also in this case, the presence of voids between the polymer and the transverse fibers of the flax composite, differently from the jute composite, clearly evidences that the polymer-fiber adhesion strongly depends on the fiber kind, although both of them have the same layout and the two composites were prepared in the same way. This result is particularly interesting since the jute fabric (as well as the flax fabric) was not chemically or physically treated, and no adhesion promoter was used. The effect of adhesion promoters on the creep resistance will be investigated in a future paper.

All of the above discussed experimental results are in perfect agreement with those coming from tensile, DMA and creep tests. The significantly better adhesion between PLA and jute fibers in comparison to flax fibers (which was already suggested by the DMA results) explains the higher creep resistance of PLA-jute composites in comparison to PLA-flax ones, although the fabrics as received showed very similar creep behaviors. On the other hand, the difference between the tensile strengths of the fabrics (i.e., about 140% higher in the flax fabric if compared to the jute one) is reduced for PLA composites (i.e., about 50% higher in the PLA-flax composite in comparison to the jute one), due to the previously discussed difference in matrix-fiber adhesion.

## 3. Materials and Methods 

The polymer matrix adopted to prepare the composites is a sample of PLA, commercialized by Natureworks (Minnetonka, MN, USA) as PLA 3100HP (melt flow index = 24 g/10 min at 210 °C/2.16 kg, melting temperature = 200 °C). Flax and jute twill weave woven fabrics, having areal density of 320 g/m^2^ and of 400 g/m^2^, respectively, were used as reinforcement. In particular, flax fabric was supplied by Lineo (Saint Martin du Tilleul, France) while jute fabric was provided by Composites Evolution (Chesterfield, UK).

In order to protect the polymer matrix by possible hydrolytic scission during processing, the PLA as well as the fabrics were dried overnight under vacuum at 90 °C, according to our previous studies [16,44].

All of the specimens for the mechanical testing were prepared by cutting them off compression molded sheets obtained by means of a Carver (Wabash, IN, USA) Laboratory press (*T* = 210 °C, *P* ≈ 250 bar, time = 4 min). In particular, each composite was prepared by stacking one fiber layer (i.e., a woven fabric) between two PLA sheets (previously prepared by compression molding as well). Table 4 reports the average values with relative deviations standard of thickness, fiber weight fraction and fiber volume fraction of neat PLA and composites, calculated on all the specimens used for the mechanical characterization (i.e., quasi-static tensile tests, dynamic mechanical tests and creep tests; the specimens were not subjected to specific conditioning treatments before testing).

Quasi-static tensile tests were carried out on dog-bone samples according to ASTM D 3039 standard, by using a Z005 universal testing machine (Zwick-Roell, Ulm, Germany) equipped with a load cell of 5 kN. At least seven samples for each system were tested. In particular, the width and the length of the narrow section were 6 mm and 33 mm, respectively. The grip distance was 65 mm, and the crosshead speed was set equal to 2 mm/min. Tensile tests were performed also on dry fabrics according to ASTM D 5035-95. 

Dynamic mechanical analysis (DMA) in tensile mode was performed using a DMA+150 apparatus (Metravib, Limonest, France) equipped with a load cell of 150 N, according to ASTM D 4065 standard. Three prismatic samples of size 46 mm × 5 mm were tested for each composite. Temperature scanning was performed from room temperature to 120 °C by setting the heating rate equal to 2 °C/min at an oscillation frequency of 1 Hz.

Creep tests were conducted on both the composites and the woven fabrics, using a new dedicated apparatus (IDEA, Termini Imerese, Italy), consisting in an oven, equipped with four extensometers, directly connected to mobile clamps and to weight holders. The samples are mounted between the two clamps and the tests start when suitable weights are applied at the end of the extensometer, as described in our previous works [42,45]. More in details, two different temperatures (40 and 60 °C) were applied, under constant tensile stress (i.e., 3 MPa) for one week. At least four samples for each system and load condition were tested. Reproducibility was satisfactory (±6%). Similarly to quasi-static characterization, creep tests was performed also on fabrics as received in order to investigate the effect of the fabric viscoelasticity on the creep behavior of the resulting composites.

Scanning electron microscopy (SEM) analysis was performed on the tensile-fractured surfaces of composites by using a FEI (Hillsboro, OR, USA) Quanta 200F scanning electron microscope. Before analysis, each sample was sputter-coated with a thin layer of gold to avoid electrostatic charging under the electron beam.

## 4. Conclusions

In this work, the creep behavior of PLA composites reinforced with flax and jute twill weave woven fabrics manufactured through compression molding technique was investigated. The behavior of the prepared systems was also compared through quasi-static tensile tests and dynamic-mechanical tests. The experimental results clearly pointed out that the presence of the reinforcing natural-fiber fabrics dramatically improves the creep resistance of the obtained composite, in comparison to the neat PLA. In particular, jute composite showed far better behavior under creep conditions in comparison to flax composite, and this was mainly attributed to a better fiber-matrix adhesion, as further proved by DMA and SEM analyses. On the other hand, tensile behavior was better upon using flax fabric as reinforcement; this was explained by considering the higher tensile resistance of the flax fabric itself in comparison to the jute one, although the better jute-PLA adhesion degree reduced the performance gap between the two composites, if compared to the one found between the two fabrics as received. 

## Figures and Tables

**Table 1:** Tensile properties of the investigated composites and fabrics.

Sample	Elastic Modulus, GPa	Tensile Strength, MPa	Elongation at Break, %
PLA	2.40 ± 0.20	49.5 ± 5.4	2.4 ± 1.4
PLA-Flax	4.26 ± 0.43	75.7 ± 7.4	2.8 ± 0.4
PLA-Jute	4.27 ± 0.25	50.0 ± 2.1	1.7 ± 0.2
Flax fabric	-	32.2 ± 0.3	4.3 ± 0.3
Jute fabric	-	13.3 ± 0.7	5.6 ± 0.3

**Table 2:** Dynamic mechanical properties of the investigated samples.

Sample	*E’* (30°C), GPa	*T*_1/2_, °C	*T*_g_, °C	Tanδ Peak Height
PLA	3.06 ± 0.07	56 ± 0.4	64.6 ± 0.4	2.38 ± 0.02
PLA-Flax	6.32 ± 0.18	57.8 ± 0.2	64 ± 0.5	0.51 ± 0.01
PLA-Jute	5.40 ± 0.09	61.5 ± 0.1	64.8 ± 0.1	0.43 ± 0.01

**Table 3:** Ratios between normalized deformation of the composite and normalized deformation of neat PLA at specific time.

Temperature, °C	Sample	8,000 s	65,000 s	250,000 s	650,000 s
40	PLA-Flax	0.98	0.95	0.92	0.89
	PLA-Jute	0.94	0.81	0.74	0.72
60	PLA-Flax	0.64	0.66	0.61	0.56
	PLA-Jute	0.23	0.22	0.20	0.19

**Table 4:** Thickness and fiber fractions of the composites.

Sample	Thickness, mm	Fiber Weight Fraction, %	Fiber Volume Fraction, %
PLA	0.740 ± 0.020	-	-
PLA-Flax	0.698 ± 0.016	38.2 ± 1.6	30.6 ± 0.4
PLA-Jute	0.750 ± 0.021	44.4 ± 0.9	41.1 ± 1.2
